# Impact of *IGF2BP2* genetic variants and expression levels with the progression of endometrial cancer

**DOI:** 10.7150/ijms.132120

**Published:** 2026-05-01

**Authors:** Chieh-Yi Kan, Yi-Hung Sun, Chung-Yuan Lee, Shih-Chi Su, Cheng-Chang Chang, Chih-Hsin Tang, Hsiao-Ju Chu, Po-Hui Wang, Shun-Fa Yang

**Affiliations:** 1 Department of Obstetrics and Gynecology, Chi-Mei Medical Center, Tainan, Taiwan.; 2 Department of Obstetrics and Gynecology, Pingtung Veterans General Hospital, Pingtung, Taiwan.; 3 Department of Nursing, Meiho University, Pingtung, Taiwan.; 4 College of Pharmacy and Health care, Tajen University, Pingtung, Taiwan.; 5 Department of Obstetrics and Gynecology, Chiayi Chang Gung Memorial Hospital Chiayi, Taiwan.; 6 Department of Nursing, Chang Gung University of Science and Technology, Chiayi Campus, Chiayi, Taiwan.; 7 Whole-Genome Research Core Laboratory of Human Diseases, Chang Gung Memorial Hospital, Keelung, Taiwan.; 8 Department of Medical Biotechnology and Laboratory Science, College of Medicine, Chang Gung University, Taoyuan, Taiwan.; 9 School of Medicine, Chung Shan Medical University, Taichung, Taiwan.; 10 Department of Obstetrics and Gynecology, Chung Shan Medical University Hospital, Taichung, Taiwan.; 11 Department of Pharmacology, School of Medicine, China Medical University, Taichung, Taiwan.; 12 Department of Medical Laboratory Science and Biotechnology, Asia University, Taichung, Taiwan.; 13 Chinese Medicine Research Center, China Medical University, Taichung, Taiwan.; 14 Institute of Medicine, Chung Shan Medical University, Taichung, Taiwan.; 15 Department of Medical Research, Chung Shan Medical University Hospital, Taichung, Taiwan.

**Keywords:** insulin-like growth factor 2 mRNA binding protein 2, endometrial cancer, single-nucleotide polymorphisms

## Abstract

Endometrial cancer (EC) is the most common gynecological cancer among women in high-income countries, and its intricate etiology involves a composite of genetic factors. Insulin-like growth factor 2 mRNA binding protein 2 (IGF2BP2) targets distinct types of RNA species to orchestrate tumor cell metabolism, invasion, and metastasis. Nevertheless, the impact of *IGF2BP2* gene polymorphisms on the development of EC remains poorly understood. Here, to clarify the genetic association between *IGF2BP2* and EC development, genotyping of three single-nucleotide polymorphisms (SNPs) of* IGF2BP2* gene, including rs11705701, rs4402960, and rs1470579, were conducted in 190 patients and 295 cancer-free women. We found that none of these three loci was in association with the risk of developing EC. Further evaluation on their correlation with key clinicopathological features revealed that EC patients carrying at least one minor allele of rs1470579 (C) or rs4402960 (T) tend to develop cervical invasion more frequently than do those homozygous for the major allele. Analysis of omics data available in the Genotype-Tissue Expression (GTEx) Portal and The Cancer Genome Atlas (TCGA) indicated that genotypes of rs1470579 and rs4402960 are associated with elevated IGF2BP2 expression, and IGF2BP2 induction in tumors was linked to progression to advanced disease and poor survival in patients with uterine corpus endometrial carcinoma. These results suggest that alterations in IGF2BP2 levels attributed by genetic variants may influence EC aggressiveness.

## Introduction

Endometrial cancer (EC) is a malignancy originating within the inner epithelial lining of the uterus and ranked the most common gynecological cancer among women in high-income countries 1, 2. Unfortunately, recent epidemiological findings reveal an upward trend in both incidence and mortality of this disease 1, 3. Such high occurrence and death rates are largely due to the heterogeneity of its interconnected risk factors. It is recognized that age (≥55 years), obesity, and prolonged exposure to excessive unopposed estrogen can drive endometrial tumorigenesis 4. In addition, a genetic predisposition to EC was previously observed in women with two autosomal dominant diseases, Lynch syndrome and Cowden syndrome 4, indicating an involvement of inherited risks in EC development. As Lynch syndrome is characterized by the presence of germline pathogenic mutations in mismatch repair (MMR) genes 5, the much rarer Cowden syndrome is caused by germline mutations in a tumor-suppressor gene, *PTEN* 6. Moreover, current genome-wide association studies have identified a variety of EC-associated genetic loci to interpret cancer etiology, biology, and therapy 7, 8, suggesting that a large portion of EC genetic heritability remains unexplored.

Insulin-like growth factor 2 mRNA binding protein 2 (IGF2BP2) was initially discovered as an interacting partner of insulin-like growth factor 2 (*IGF2*) gene transcripts 9. Its major function is known to involve the modulation of RNA localization, stability, and translation 10. A key impact of IGF2BP2 on metabolic regulation and insulin resistance was noticed via a post-transcriptional control of multiple genes 11. Besides interactions with numerous mRNA molecules, IGF2BP2 also binds to long noncoding RNAs that are epitranscriptionally modified with N6-methyladenosine (m6A) 12. By virtue of its broad range of interacting RNA partners, IGF2BP2 is shown to mediate a variety of cellular responses that lead to many pathogenic conditions, such as metabolic syndrome and cancer 13. Concordantly, recent association studies employing targeted gene approaches have uncovered a link of *IGF2BP2* gene polymorphisms to diabetes and various cancers 14-17. In several types of malignancies, a correlation between IGF2BP2 induction and poor prognosis has been observed 18-23, indicating a role of IGF2BP2 in promoting tumorigenesis. Mechanistically, IGF2BP2 targets distinct types of RNA species within different pathways to modulate tumor cell invasion, apoptosis, metastasis, and metabolism 24. In a recent study on exploring the molecular mechanism of an ATP-dependent RNA helicase protein, DDX17 (DEAD-box helicase 17), in regulating EC progression, IGF2BP2 was demonstrated to specifically recognize and stabilize m6A-modified *DDX17* gene transcripts, resulting in enhanced tumor-regulatory effects 25. Additionally, a circular RNA, circCHD7, was enriched through IGF2BP2-mediated m6A modification to increase the mRNA stability of platelet-derived growth factor receptor beta (*PDGFRB*) gene, thereby promoting the proliferation of EC cells 26. As emerging roles of IGF2BP2 in EC progression were revealed, the effect of *IGF2BP2* gene polymorphisms on the development of EC remains elusive. Here, we performed a case-control survey to examine a genetic association of *IGF2BP2* single-nucleotide polymorphisms (SNPs) with endometrial tumorigenesis.

## Materials and Methods

### Study cohort

In this targeted gene study, 190 EC patients were recruited in Chung Shan Medical University Hospital (Taichung, Taiwan) and the National Biobank Consortium of Taiwan (NBCT) (Miaoli, Taiwan). Cancer diagnosis was confirmed histologically, and cases with type I (endometrioid adenocarcinoma) and type II (non-endometrioid adenocarcinoma) EC were enrolled and staged clinically based on the International Federation of Gynecology and Obstetrics (FIGO) Staging System 27 at the time of disease diagnosis. In addition, 295 women who had neither history of cancer of any site nor clinical evidence of endometrial pathologies were recruited from the Chung Shan Medical University Hospital for comparisons. This study was approved by the institutional review board of Chung Shan Medical University Hospital (No: CS1-23140), with the informed written consent provided by all subjects at enrollment.

### Genotyping of IGF2BP2 single-nucleotide polymorphisms (SNPs)

Three SNPs (rs1470579, rs4402960, and rs11705701) of *IGF2BP2* gene previously identified to confer the susceptibility to diverse medical conditions 14-17, 28, 29 were selected and genotyped in this survey. Genomic DNA of whole blood specimens was extracted by using a QIAamp DNA Blood Mini Kit (Qiagen, Valencia, CA, USA)^30^. Allelic discrimination for three loci of *IGF2BP2* gene was determined by using the TaqMan® Allelic Discrimination Demonstration Kit (Thermo Fisher Scientific) with a StepOnePlus Real-Time PCR System (Applied Biosystems, Foster City, CA, USA).

### Endometrial cancer cell culture and IGF2BP2 mRNA expression

The human EC cell lines KLE, AN3CA, and HEC-1-A were obtained from the American Type Culture Collection (ATCC; Manassas, VA, USA), while the human EC cell lines SNG-M, HEC-265, and HEC-50B were obtained from the Japanese Collection of Research Bioresources (JCRB; Osaka, Japan). Each culture medium was supplemented with 10% fetal bovine serum and 1% penicillin-streptomycin-glutamine (Life Technologies). Cells were incubated at 37°C in an atmosphere of 5% CO₂ and 95% air. The IGF2BP2 mRNA levels of EC cell lines was evaluated with a real-time PCR assay as described previously 31.

### Statistical analysis

Comparison of age between EC patients and non-cancer controls was performed by using Fisher's exact test. The adjusted odds ratios (AORs) together with the 95% confidence intervals (CIs) for the association between EC risk and genotype frequency was calculated by multiple logistic regression models after controlling for age. The survival of patients assigned by gene expression data retrieved from The Cancer Genome Atlas (TCGA) was assessed with a Kaplan-Meier plotter and compared by using the log-rank test. The differences in IGF2BP2 transcript levels from the uterine corpus endometrial carcinoma dataset of TCGA were compared by Student's t test. Significant association between genotypes and IGF2BP2 expression levels in the Genotype-Tissue Expression (GTEx) portal was determined by one-way ANOVA. Data were analyzed by using SAS statistical software (Version 9.1, 2005; SAS Institute Inc., Cary, NC). A *p* value < 0.05 was considered significant.

## Results

### Cohort characteristics

In this study, 190 EC patients were enrolled to interrogate the genetic association of *IGF2BP2* gene polymorphisms with the risk of developing endometrial neoplasm. To exclude potential confounding effects, we recruited 295 cancer-free women with matched chronological age as the control group (Table 1). Among 190 EC patients, 157 (82.6%) and 33 (17.4%) suffered from early-stage and advanced cancer, respectively. Myometrial, cervical, and vascular invasion were confirmed in 75.3%, 20%, and 13.7% of cases, respectively.

### IGF2BP2 gene polymorphisms are associated with EC progression

To clarify whether *IGF2BP2* confers a genetic susceptibility to EC, three disease-associated SNPs (rs1470579, rs4402960, and rs11705701) were examined to determine their correlation with the development or progression of EC in this survey. Our result demonstrated that none of these* IGF2BP2* SNPs was correlated with the risk of developing EC in our cohorts (Table 2). To further explore the potential association of three SNPs with EC progression, we subsequently evaluated their genotype distribution between different patient groups assigned by key clinicopathological features. Our data showed that patients who bear at least one minor allele of rs1470579 (C) or rs4402960 (T) have tumors spread from the uterine lining into the cervical stroma more frequently than those who are homozygous for the major allele after controlling for age and clinicopathological parameters (AC+CC vs AA for rs1470579; AOR, 2.385; 95% CI, 1.002-5.675; *p*=0.049) (GT+TT vs GG for rs4402960; AOR, 2.543; 95% CI, 1.067-6.064; *p*=0.035) (Table 3-4). Nevertheless, such genetic association with cervical invasion of EC was not observed among patients of different rs11705701 genotypes (Table 5). These findings indicate an effect of *IGF2BP2* polymorphic alleles on promoting the aggressiveness of endometrial tumor cells.

### Functional relevance of rs1470579 and rs4402960 in EC progression

**Figure 1:**
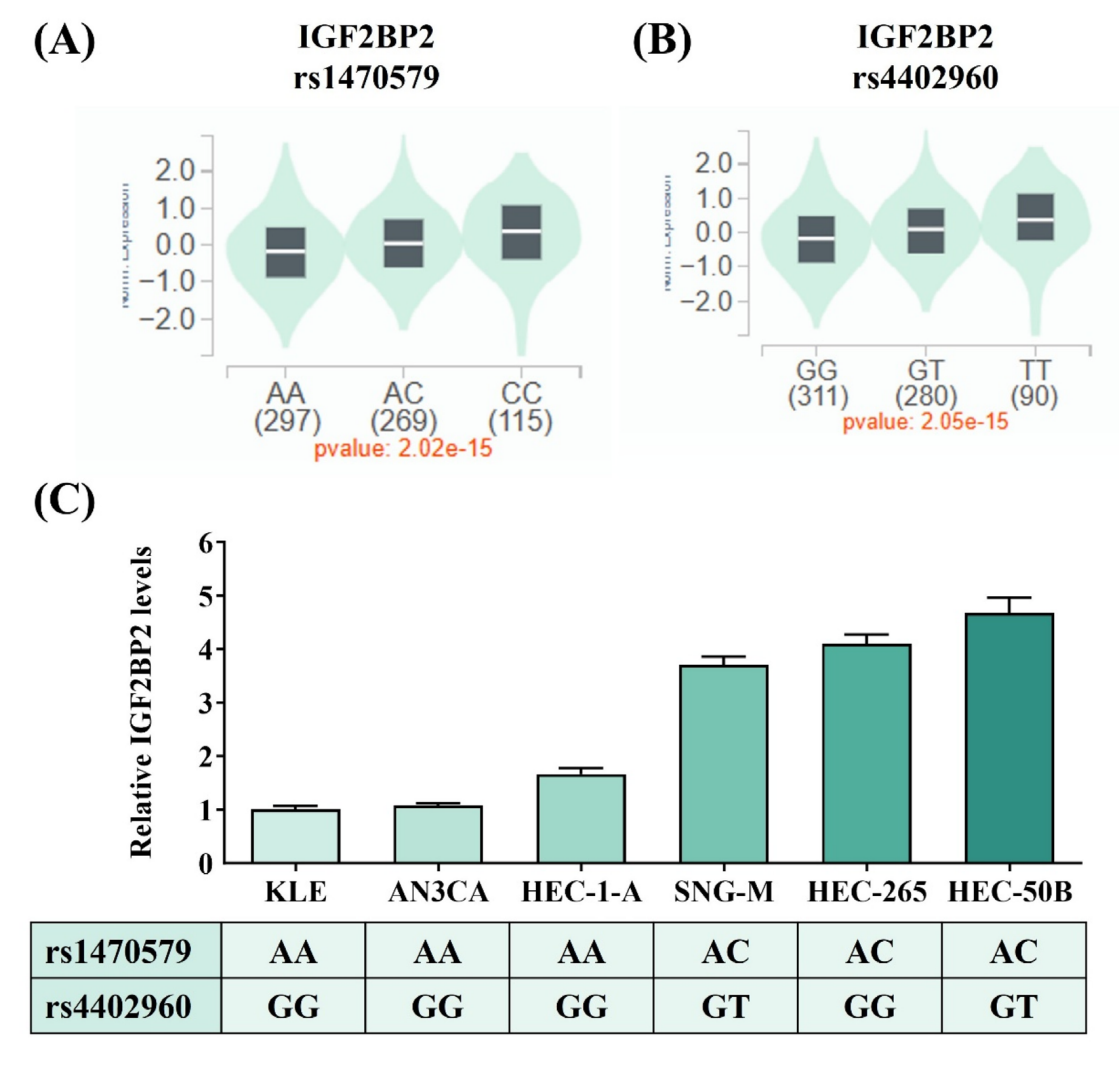
** IGF2BP2 SNPs, rs1470579 and rs4402960**,** influence the expression levels of IGF2BP2. (A-B)** Genotypes of rs1470579 and rs4402960 are associated with an elevation in IGF2BP2 expression in thyroid tissues based on data from the GTEx portal. **(C)** IGF2BP2 mRNA expression levels were quantified using real-time PCR in six endometrial cancer cell lines with known genotypes for rs1470579 and rs4402960. *p* values are calculated with one-way ANOVA.

**Figure 2:**
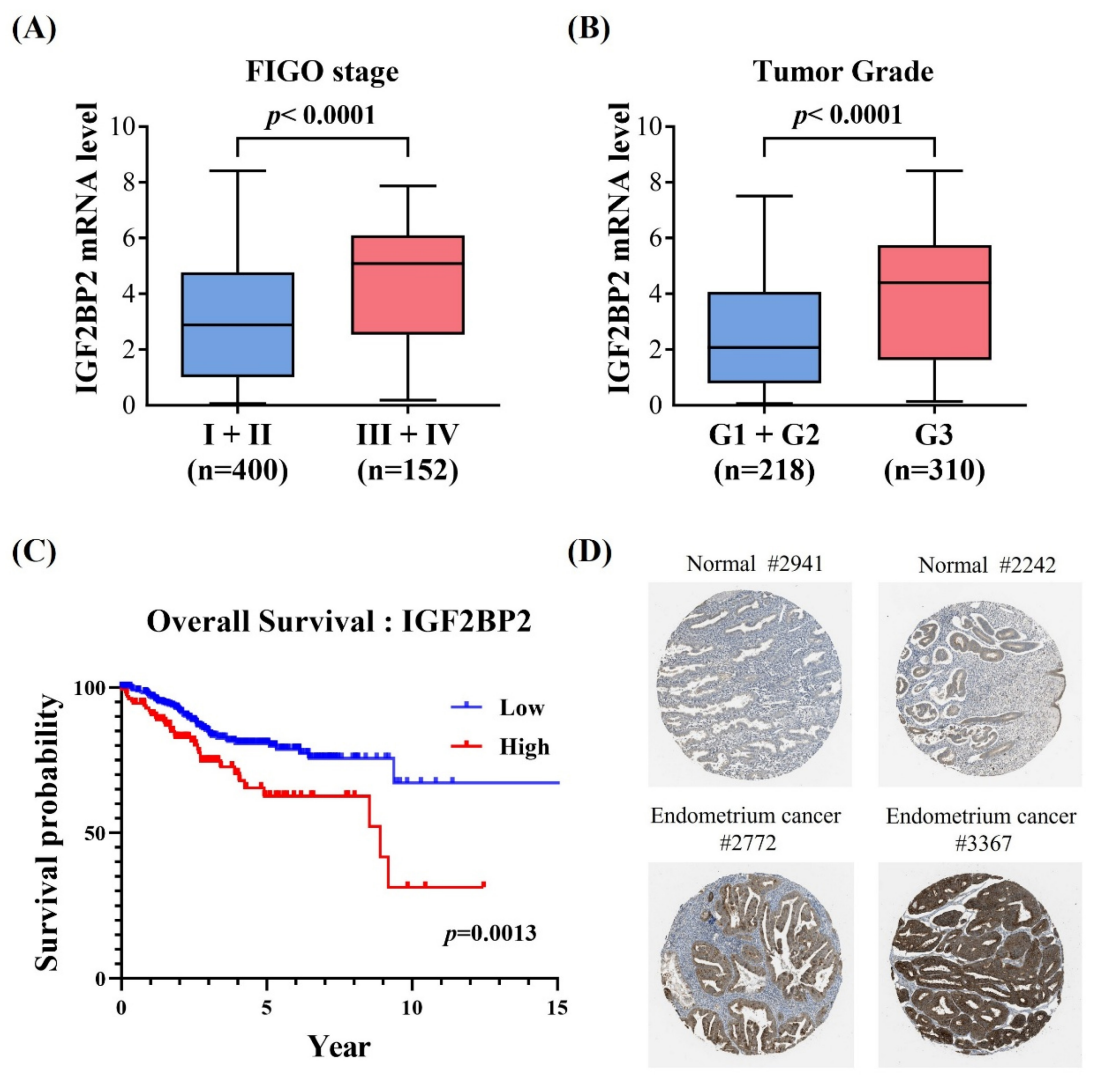
** Clinical relevance of IGF2BP2 expression in endometrial carcinoma. (A-C)** Correlation of augmented IGF2BP2 levels with advanced FIGO stage (III-IV) **(A)**, higher FIGO tumor grade (G3) **(B)**, and poorer overall survival **(C)**. **(D)** The Human Protein Atlas project provides representative immunohistochemical images of IGF2BP2 expression in endometrial carcinoma tissues compared with normal endometrium.

Considering that two intronic SNPs, rs1470579 and rs4402960, were detected in association with EC progression, we next attempted to infer tentative functionality of these two loci through surveying data from public resources. Analysis of GTEx data revealed that carrying the minor allele of rs1470579 and rs4402960 is linked to an increase in expression levels of IGF2BP2 in thyroid tissues (Figures 1A-1B), implicating these two EC-associated SNPs as cis-expression quantitative trait loci (cis-eQTLs). Consistent with the GTEx findings, real-time PCR analysis showed that all three endometrial cancer cell lines carrying the rs1470579 AC genotype (SNG-M, HEC-265, and HEC-50B) exhibited higher IGF2BP2 mRNA levels (Figure 1C). Moreover, expression analysis revealed that the minor allele (T) of rs4402960 is associated with increased IGF2BP2 mRNA levels in the SNG-M and HEC-50B endometrial cancer cell lines (Figure 1C). Furthermore, TCGA analysis demonstrated that elevated IGF2BP2 expression in uterine corpus endometrial carcinoma was associated with advanced FIGO stage (III-IV) (Figure 2A), advanced FIGO tumor grade (G3) (Figure 2B), and poorer patient survival (Figure 2C). Additionally, immunohistochemical (IHC) data from the Human Protein Atlas revealed strong IGF2BP2 expression in EC tissues but weak expression in normal endometrium (Figure 2D). These results underpin the observation that allele-specific enhancement of IGF2BP2 levels may contribute to EC progression.

## Discussion

The development and aggressiveness of EC entail a list of cancer hallmark events that are directed by both genetic and environmental parameters. Here, through conducting a case-control study, we detected an association of *IGF2BP2* variations (rs1470579 and rs4402960) with cervical invasion of EC. Moreover, these two EC-associated loci may function as cis-eQTLs that enhance IGF2BP2 expression, which is associated with poor patient survival and disease progression to advanced stage and higher tumor grade.

Unlike rs11705701 that is positioned in the 5' promoter region and exhibits no association with EC, two EC-associated SNPs (rs1470579 and rs4402960) reported here are located in the second intron of *IGF2BP2* gene and in strong linkage disequilibrium with each other 32, which to some degree accounts for their effects on configuring similar patterns of disease susceptibility in our findings. Both rs1470579 and rs4402960 have been extensively studied and proved as genetic susceptibility loci to diabetes mellitus (DM) across multiple ethnicities 32, 33. Since schizophrenia is in a robust association with DM 34, rs1470579 and rs4402960 have also been demonstrated as genetic biomarkers for schizophrenia 29, 35, suggesting shared genetic risks and disclosing a number of overlapped predisposition loci between two diseases. In addition, recent association studies have linked *IGF2BP2* gene polymorphisms to various cancers, both positively and negatively. Specifically, a tumor-suppressive effect on triple negative breast cancer 36 and non-small-cell lung cancer 37 was associated with the minor allele of rs1470579 and rs4402960. On the contrary, male patients with prostate cancer 17 and oral squamous cell carcinoma 16 had an increased risk of cancer metastasis while carrying the minor allele of rs1470579 or rs4402960, revealing their effect on tumor promotion. In our study, EC patients with at least one minor allele of rs1470579 or rs4402960 tended to develop cervical invasion more frequently than those homozygotes for the major allele, indicating that such discrepancy in dual roles of *IGF2BP2* variants on tumorigenesis is likely related to tissue specificity but not gender.

Although some efforts on exploring the biological functions of *IGF2BP2* rs1470579 and rs4402960 have been made, how these two disease-associated loci take on their pathogenic roles remains undefined. A previous in silico analysis predicted that *IGF2BP2* rs1470579 and rs4402960 might create or alter an exonic splicing silencer and/or an axonic splicing enhancer 35, suggesting their potential to affect splicing events. To data, three IGF2BP2 protein isoforms have been identified in humans. In addition to the full-length isoform with a molecular weight of 66 kDa, human *IGF2BP2* gene encodes a splicing variant (62 kDa) with a deletion between the K homology (KH) 2 and KH3 domain 38 as well as a shorter (58 kDa) version lacking an N-terminal RNA recognition motif 39, adding another dimension of structural complexity and RNA binding capacity, specificity, and versatility 40. Intriguingly, biochemical quantification of IGF2BP2 isoforms in visceral adipose tissues indicated that the minor allele of rs4402960 was associated with reduced amounts of the shorter isoform (58 kDa) and increased levels of the full-length isoform (66 kDa) of the IGF2BP2 protein in non-obese individuals 28, implicating a functional role of rs4402960 in influencing the affinity of IGF2BP2 with its interacting RNA partners.

Besides alterations in its RNA binding specificity and versatility, rs1470579 and rs4402960 may serve as cis-eQTLs to orchestrate the expression of IGF2BP2 and/or other proteins encoded by genes nearby the second intron of *IGF2BP2* gene. Consistent with our observation that the minor allele of rs1470579 and rs4402960 is linked to IGF2BP2 induction in thyroid tissues, non-obese individuals homozygous for the minor allele of rs4402960 had higher levels of IGF2BP2 mRNA in the adipose tissue than those with other genotypes 28. In addition, it has been proposed that expression of several genes that are implicated in insulin activities and also located in proximity to *IGF2BP2* gene, such as protein phosphatase 1 regulatory subunit 2 (*PPP1R2*), mitogen-activated protein kinase kinase kinase 13 (*MAP3K13*), lipase H (*LIPH*), diacylglycerol kinase g-1 (*DGKG*), a-2-HS-glycoprotein (*AHSG*), and the insulin-sensitizing adipokine adiponectin (*ADIPOQ*) could be regulated by these two EC-associated SNPs to cause diabetes 41. Collectively, our data, together with the results from others, indicate that tumors spread from the uterine lining into the cervical stroma in EC patients could be attributed by fluctuations in IGF2BP2 levels due to gene polymorphisms.

In this survey, we observed an association of *IGF2BP2* gene polymorphisms with EC spread to the cervical stroma; however, extra work is required to tackle several study limitations. One weakness is that this study comprises a modest size of study cohorts, which likely restrain the statistical power and present challenges with regard to result generalizability. Considering that a correlation between rs4402960 and the risk of developing EC was observed on the borderline (*p*=0.090), we acknowledge that many more significant genetic associations are presumably to be detected in a larger sample size. Another caveat is that the influence of *IGF2BP2* gene polymorphisms on the risk of developing EC may be underestimated due to disease phenotype heterogeneity (e.g., Type I vs Type II, molecular subtypes). Additional stratification analysis may address this issue. Moreover, the underlying mechanism through which polymorphic alleles of rs1470579 and rs4402960 mediate promotive effects on EC metastasis remains an open question. Whether the genetic variations affect the expression of IGF2BP2 isoforms or RNA binding capacity, specificity, and versatility needs further investigations. Furthermore, the GTEx-based expression analysis was derived from thyroid tissue, which may not accurately reflect gene regulation in endometrial tissue and therefore limits the direct relevance of these findings to endometrial cancer.

In conclusion, we detected a correlation between *IGF2BP2* SNPs (rs1470579 and rs4402960) and cervical invasion of EC. Enhanced IGF2BP2 expression is associated with poor survival and disease progression to advanced stage in EC patients. Our results uncover a novel genetic association of *IGF2BP2* variants with EC progression.

## Figures and Tables

**Table 1:** The distributions of demographical characteristics in 295 controls and 190 patients with endometrial cancer.

Variable	Controls (N=295)	Patients (N=190)	*p* value
Age (yrs)			
≤ 55	147 (49.8%)	80 (42.1%)	*p* = 0.096
> 55	148 (50.2%)	110 (57.9%)	
**FIGO stage**			
I+II		157 (82.6%)	
III+IV		33 (17.4%)	
**FIGO grade**			
G1		74 (39.0%)	
G2		88 (46.3%)	
G3		23 (12.1%)	
Unknown		5 (2.6%)	
**Lymph node involvement**			
No		165 (86.8%)	
Yes		24 (12.7%)	
Unknown		1 (0.5%)	
**Myometrial invasion**			
No		28 (14.7%)	
Yes		143 (75.3%)	
Unknown		19 (10.0%)	
**Cervical invasion**			
No		141 (74.2%)	
Yes		38 (20.0%)	
Unknown		11 (5.8%)	
**Vascular invasion**			
No		160 (84.2%)	
Yes		26 (13.7%)	
Unknown		4 (2.1%)	

**Table 2:** The adjusted odds ratio (AOR) and 95% confidence interval (CI) for the association between endometrial cancer risk and *IGF2BP2* genotypic frequencies.

Variable	Controls (N=295) (%)	Patients (N=190) (%)	AOR (95% CI)	*p* value
**rs1470579**				
AA	167 (56.6%)	98 (51.6%)	1.000 (reference)	
AC	108 (36.6%)	80 (42.1%)	1.245 (0.849-1.826)	0.263
CC	20 (6.8%)	12 (6.3%)	1.027 (0.480-2.195)	0.946
AC+CC	128 (43.4%)	92 (48.4%)	1.211 (0.839-1.748)	0.307
**rs4402960**				
GG	174 (59.0%)	99 (52.1%)	1.000 (reference)	
GT	101 (34.2%)	81 (42.6%)	1.394 (0.950-2.046)	0.090
TT	20 (6.8%)	10 (5.3%)	0.883 (0.396-1.965)	0.760
GT+TT	121 (41.0%)	91 (47.9%)	1.310 (0.906-1.893)	0.151
**rs11705701**				
GG	172 (58.3%)	107 (56.3%)	1.000 (reference)	
GA	106 (35.9%)	72 (37.9%)	1.086 (0.738-1.597)	0.676
AA	17 (5.8%)	11 (5.8%)	1.013 (0.456-2.250)	0.976
GA+AA	123 (41.7%)	83 (43.7%)	1.076 (0.743-1.557)	0.699

The adjusted odds ratio (AOR) with their 95% confidence intervals were estimated by multiple logistic regression models after controlling for age.

**Table 3:** Clinical statuses and genotypic frequencies of *IGF2BP2* rs1470579 in 190 endometrial cancers.

	*IGF2BP2* rs1470579			
Variable	AA (N=98)	AC + CC (N=92)	AOR (95% CI)	*p* value
**FIGO stage**				
Stage I+II	79 (80.6%)	78 (84.8%)	1.000 (reference)	0.205
Stage III+IV	19 (19.4%)	14 (15.2%)	0.777 (0.527-1.148)	
**FIGO grade^a^**				
G1	41 (41.8%)	33 (37.9%)	1.000 (reference)	0.967
G2+G3	57 (58.2%)	54 (62.1%)	1.007 (0.714-1.421)	
**Lymph node involvement^a^**				
No	86 (87.8%)	79 (86.8%)	1.000 (reference)	0.438
Yes	12 (12.2%)	12 (13.2%)	1.733 (0.432-6.950)	
**Myometrial invasion^a^**				
No	17 (18.7%)	11 (13.8%)	1.000 (reference)	0.361
Yes	74 (81.3%)	69 (86.3%)	1.526 (0.616-3.779)	
**Cervical invasion^a^**				
No	80 (86.0%)	61 (70.9%)	1.000 (reference)	0.049*
Yes	13 (14.0%)	25 (29.1%)	**2.385 (1.002-5.675)**	
**Vascular invasion^a^**				
No	84 (86.6%)	76 (85.4%)	1.000 (reference)	0.845
Yes	13 (13.4%)	13 (14.6%)	0.896 (0.296-2.709)	

The adjusted odds ratio (AOR) and 95% confidence intervals (CIs) were calculated using multiple logistic regression models after controlling for age and clinicopathological parameters. * *p* value < 0.05 as statistically significant. ^a^ Cases with unknown data points in Table 1 are excluded from statistical analysis.

**Table 4:** Clinical statuses and genotypic frequencies of *IGF2BP2* rs4402960 in 190 endometrial cancers.

	*IGF2BP2* rs4402960			
Variable	GG (N=99)	GT + TT (N=91)	AOR (95% CI)	*p* value
**FIGO stage**				
Stage I+II	80 (80.8%)	77 (84.6%)	1.000 (reference)	0.183
Stage III+IV	19 (19.2%)	14 (15.4%)	0.766 (0.518-1.133)	
**FIGO grade^a^**				
G1	41 (41.4%)	33 (38.4%)	1.000 (reference)	0.877
G2+G3	58 (58.6%)	53 (61.6%)	0.973 (0.689-1.375)	
**Lymph node involvement^a^**				
No	87 (87.9%)	78 (86.7%)	1.000 (reference)	0.419
Yes	12 (12.1%)	12 (13.3%)	1.775 (0.441-7.140)	
**Myometrial invasion^a^**				
No	18 (19.6%)	10 (12.7%)	1.000 (reference)	0.172
Yes	74 (80.4%)	69 (87.3%)	1.902 (0.756-4.785)	
**Cervical invasion^a^**				
No	81 (86.2%)	60 (70.6%)	1.000 (reference)	0.035*
Yes	13 (13.8%)	25 (29.4%)	**2.543 (1.067-6.064)**	
**Vascular invasion^a^**				
No	85 (86.7%)	75 (85.2%)	1.000 (reference)	0.912
Yes	13 (13.3%)	13 (14.8%)	0.939 (0.310-2.847)	

The adjusted odds ratio (AOR) and 95% confidence intervals (CIs) were calculated using multiple logistic regression models after controlling for age and clinicopathological parameters. * *p* value < 0.05 as statistically significant. ^a^ Cases with unknown data points in Table 1 are excluded from statistical analysis.

**Table 5:** Clinical statuses and genotypic frequencies of *IGF2BP2* rs11705701 in 190 endometrial cancers.

	*IGF2BP2* rs11705701			
Variable	GG (N=107)	GA + AA (N=83)	AOR (95% CI)	*p* value
**FIGO stage**				
Stage I+II	88 (82.2%)	69 (83.1%)	1.000 (reference)	0.422
Stage III+IV	19 (17.8%)	14 (16.9%)	0.857 (0.588-1.249)	
**FIGO grade^a^**				
G1	44 (41.9%)	30 (37.5%)	1.000 (reference)	0.593
G2+G3	61 (58.1%)	50 (62.5%)	1.098 (0.779-1.548)	
**Lymph node involvement^a^**				
No	94 (87.9%)	71 (86.6%)	1.000 (reference)	0.701
Yes	13 (12.1%)	11 (13.4%)	1.302 (0.338-5.011)	
**Myometrial invasion^a^**				
No	18 (18.7%)	10 (13.3%)	1.000 (reference)	0.418
Yes	78 (81.3%)	65 (86.7%)	1.448 (0.591-3.548)	
**Cervical invasion^a^**				
No	83 (83.0%)	58 (73.4%)	1.000 (reference)	0.110
Yes	17 (17.0%)	21 (26.6%)	1.996 (0.856-4.654)	
**Vascular invasion^a^**				
No	91 (85.8%)	69 (86.2%)	1.000 (reference)	0.677
Yes	15 (14.2%)	11 (13.8%)	0.791 (0.262-2.385)	

The adjusted odds ratio (AOR) and 95% confidence intervals (CIs) were calculated using multiple logistic regression models after controlling for age and clinicopathological parameters. ^a^ Cases with unknown data points in Table 1 are excluded from statistical analysis.
